# Supplementary material for: Synthetic extracellular matrices with tailored adhesiveness and degradability support lumen formation during angiogenic sprouting
Source: Nat Commun. 2021 Jun 7;12:3402. doi: 10.1038/s41467-021-23644-5 (PMC8184799; doi:10.1038/s41467-021-23644-5)
Supplement: Supplementary file 1 — Supplementary Information [file 41467_2021_23644_MOESM1_ESM.pdf]

Supplementary Information

Synthetic extracellular matrices with tailored adhesiveness and degradability support lumen formation during angiogenic sprouting

Jifeng Liu[1], Hongyan Long[1], Dagmar Zeuschner[2], Andreas F. B. Räder[3], William J. Polacheck[4], Horst Kessler[3], Lydia Sorokin[5], Britta Trappmann[1*]

[1] Bioactive Materials Laboratory, Max Planck Institute for Molecular Biomedicine, Röntgenstraße 20, 48149 Münster, Germany

[2] Electron Microscopy Unit, Max Planck Institute for Molecular Biomedicine, Röntgenstraße 20, 48149 Münster, Germany

[3] Department of Chemistry, Technical University of Munich, Lichtenbergstraße 4, 85747 Garching, Germany

[4] Joint Department of Biomedical Engineering, University of North Carolina at Chapel Hill and North Carolina State University, 9206B Mary Ellen Jones Building, 116 Manning Drive, Chapel Hill, NC 27514, USA

[5] Institute of Physiological Chemistry and Pathobiochemistry and Cells in Motion Interfaculty Centre (CiMIC), University of Münster, Waldeyerstraße 15, 48149 Münster, Germany

[*] Correspondence
Britta Trappmann, Ph.D.
Max Planck Institute for Molecular Biomedicine
Röntgenstr. 20
D-48149 Münster
Germany
Phone: ++ 49-251-70365-570
Email: britta.trappmann@mpi-muenster.mpg.de

Integrin Binding Ligand	$\alpha_v\beta_3$ (nM)	$\alpha_5\beta_1$ (nM)
CGRGDS	28±3	77±8
$\alpha_v\beta_3$-Selective Ligand c[RGDfK(C)] 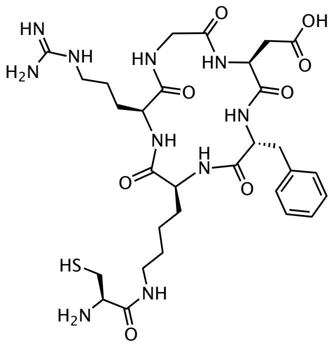	2.25±0.34	141±15
$\alpha_5\beta_1$-Selective Ligand 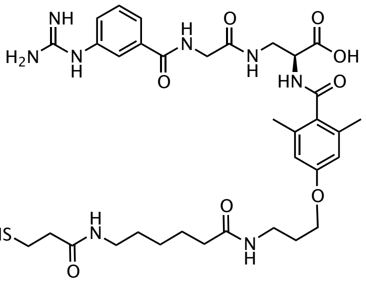	>10,000	2.3±0.02

Supplementary Table 1. Affinities of integrin binding ligands. IC_{50}-values for soluble ligands engaging integrins $\alpha_v\beta_3$ and $\alpha_5\beta_1$, obtained from literature[29].

Peptide substrate k_{cat}/K_M ($M^{-1}s^{-1}$)							
Name	Sequence	MMP-1	MMP-2	MMP-3	MMP-7	MMP-9	MT1-MMP
NCD	GPQG-IAGQ	27.4 ± 0.9	10,100 ± 400	160 ± 40	180 ± 20	8,400 ± 200	3,600 ± 200
HD	VPMS-MRGG	1,600 ± 100	24,000 ± 1000	3,900 ± 400	7,900 ± 900	51,000 ± 3000	6,100 ± 300

Supplementary Table 2. Cleavage kinetics for MMP cleavable crosslinker peptides by different MMPs. k_{cat}/K_M values for crosslinker peptides with native collagen-derived degradability (NCD) and high degradability (HD) by different MMPs were obtained from literature[33].

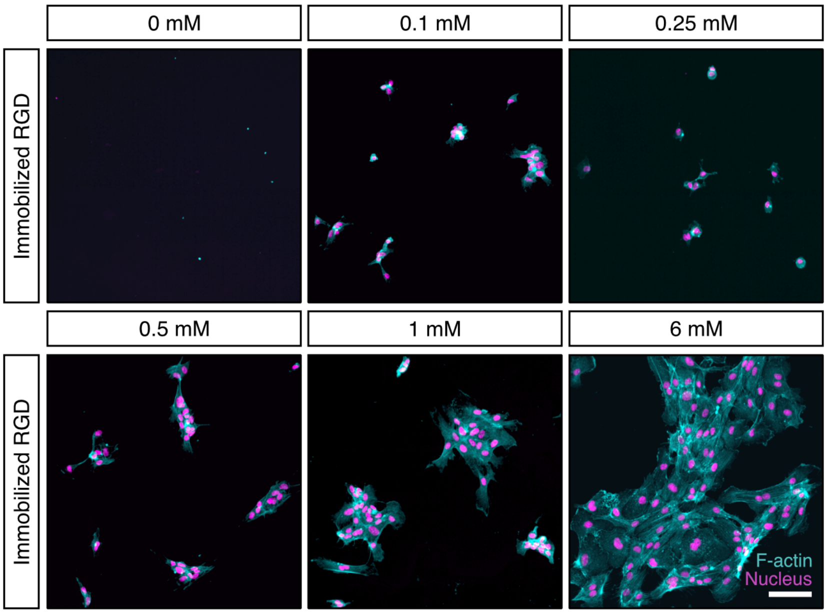

Supplementary Figure 1. Cell spreading is regulated by matrix adhesiveness. Morphology of HUVECs seeded on methacrylated dextran (DexMA) hydrogels functionalized with varying concentrations of the cell adhesive peptide CGRGDS. Composite fluorescence images of 3D projections showing F-actin (cyan) and nuclei (magenta) (*scale bar*, 100 μm).

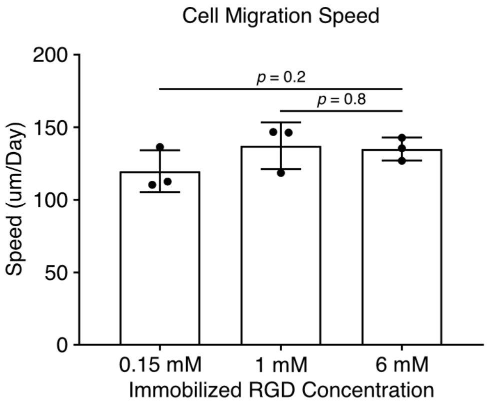

Supplementary Figure 2. Speed of endothelial cell invasion is independent of the concentration of matrix-tethered RGD. HUVECs were induced to sprout into methacrylated dextran (DexMA) hydrogels functionalized with varying concentrations of the cell adhesive peptide CGRGDS for 3.5 days. Total coupled ligand concentration was kept constant at 6 mM by adjusting with non-adhesive ligand CGRGES. Concentration of native collagen-derived degradability (NCD) crosslinker was kept constant at 30.5 mM to ensure comparable stiffness. Cell migration speed was quantified (n = 3 independent experiments). All data presented as mean ± s.d., $p < 0.05$ is considered to be statistically significant (two-tailed unpaired Student's *t*-test). Source data are provided as a source data file.

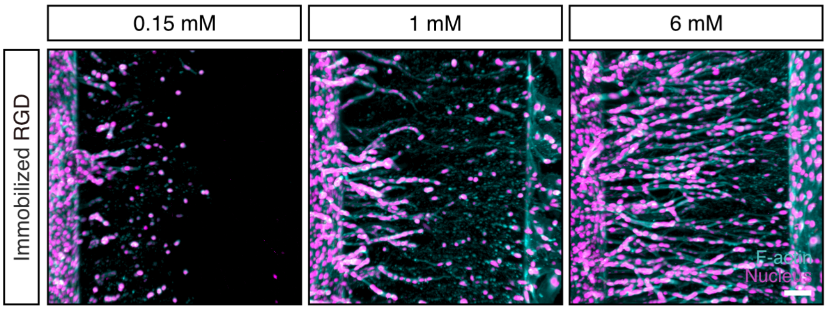

Supplementary Figure 3. Matrix adhesiveness regulates multicellularity of angiogenic sprouting after extended *in vitro* culture. HUVECs invading dextran vinyl sulfone (DexVS) hydrogels functionalized with varying concentrations of the adhesive peptide CGRGDS for 14 days. Coupled ligand concentration was kept constant at 6 mM by adjusting with non-adhesive ligand CGRGES. Concentration of native collagen-derived degradability (NCD) crosslinker was kept constant at 25.2 mM to ensure comparable stiffness. Composite fluorescence images of 3D projections showing F-actin (cyan) and nuclei (magenta) (*scale bar*, 100 μm).

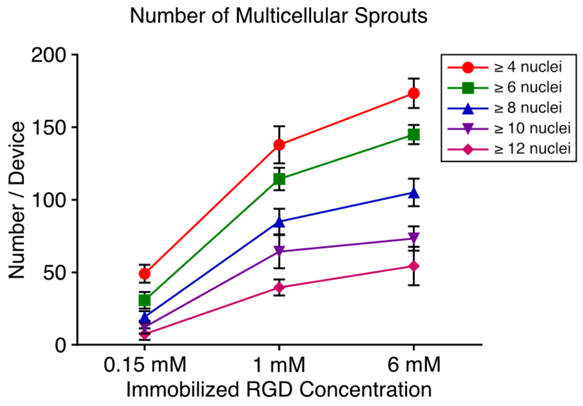

Supplementary Figure 4. Analysis of sprout multicellularity with different nuclear cutoffs. Sprout multicellularity was determined by counting the number of actin structures containing nuclei equal to or above a preset cutoff number. To examine the effect of this cutoff's value selection, data from the study determining the effect of varying concentrations of methacrylated dextran (DexMA)-tethered CGRGDS was re-analyzed using 4, 6, 8, 10 or 12 nuclei as cutoff (n = 3 independent experiments). All data presented as mean ± s.d. Source data are provided as a source data file.

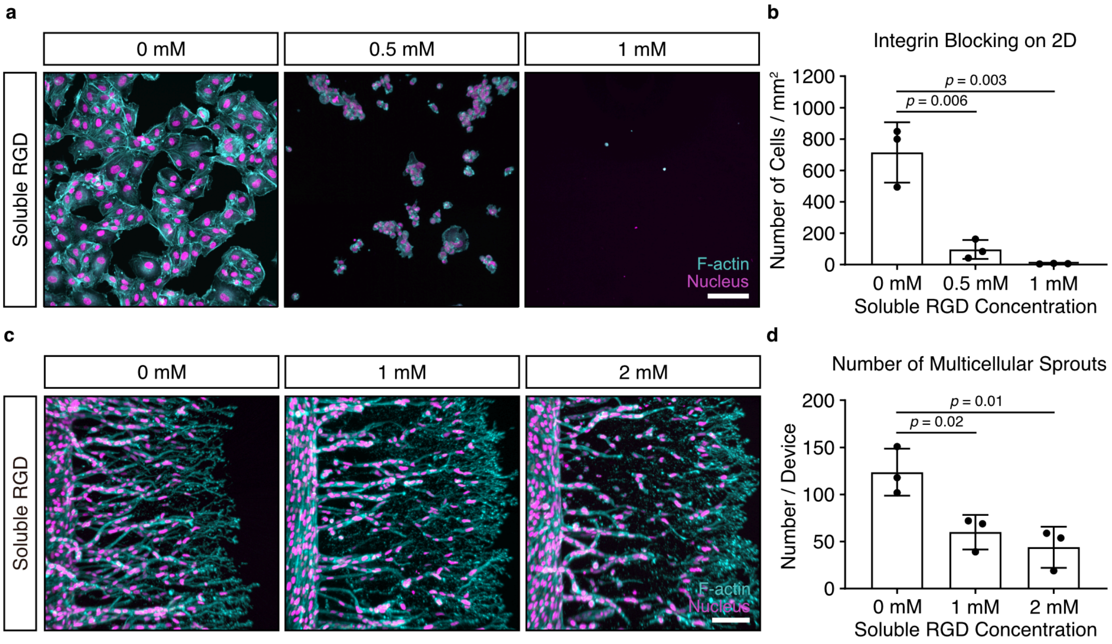

Supplementary Figure 5. Collective endothelial cell migration is inhibited by the inactivation of integrins. a HUVECs seeded on methacrylated dextran (DexMA) hydrogels functionalized with 6 mM CGRGDS were allowed to spread for 2 hours in the presence of varying concentrations of the soluble integrin inhibiting peptide GRGDS. **b** Quantification of number of HUVECs attached per mm^2 DexMA hydrogel surface as a function of soluble GRGDS concentration in the culture medium (n = 3 independent experiments). **c** HUVECs invading into DexMA hydrogels functionalized with 6 mM immobilized CGRGDS, in the presence of varying concentrations of soluble GRGDS. **d** Quantification of number of multicellular sprouts (sprouts per device possessing 6 or more nuclei) (n = 3 independent experiments). Composite fluorescence images of 3D projections showing F-actin (cyan) and nuclei (magenta) (*scale bar*, 100 μm). All data presented as mean ± s.d., $p < 0.05$ is considered to be statistically significant (two-tailed unpaired Student's *t*-test). Source data are provided as a source data file.

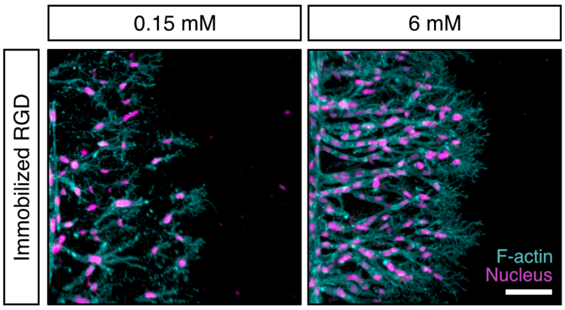

Supplementary Figure 6. Collective migration of microvascular endothelial cells is regulated by matrix adhesiveness. HMVECs invading methacrylated dextran (DexMA) hydrogels functionalized with 0.15 mM or 6 mM of the adhesive peptide CGRGDS for 3.5 days. Total coupled ligand concentration was kept constant at 6 mM by adjusting with non-adhesive ligand CGRGES. Concentration of native collagen-derived degradability (NCD) crosslinker was kept constant at 30.5 mM to ensure comparable stiffness. Composite fluorescence images of 3D projections showing F-actin (cyan) and nuclei (magenta) (*scale bar*, 100 μm).

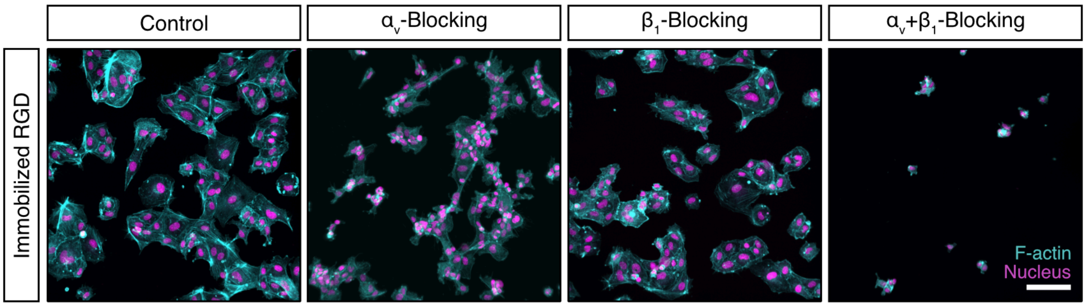

Supplementary Figure 7. RGD is not an integrin selective ligand. HUVECs were seeded on methacrylated dextran (DexMA) hydrogels functionalized with 6 mM CGRGDS and cultured for 2 hours in the presence of β_1 and α_v integrin blocking antibodies. Only if cells were treated with a combination of both antibodies, integrin activation was fully inhibited, as observed by inhibited cell spreading. Composite fluorescence images of 3D projections showing F-actin (cyan) and nuclei (magenta) (*scale bar*, 100 μm).

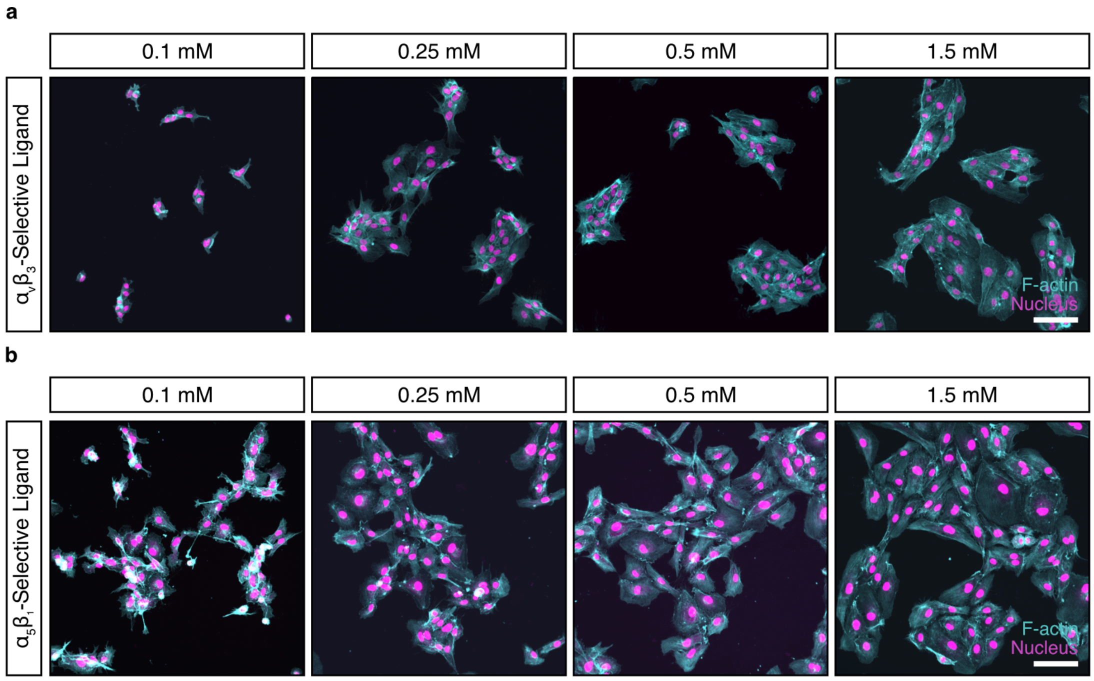

Supplementary Figure 8. Cell spreading is regulated by integrin $\alpha_5\beta_1$ and $\alpha_v\beta_3$-selective ligands. HUVECs were seeded on methacrylated dextran (DexMA) hydrogels functionalized with varying concentrations of $\alpha_v\beta_3$- (**a**) or $\alpha_5\beta_1$-selective (**b**) ligand and allowed to spread for 24 hours. Composite fluorescence images of 3D projections showing F-actin (cyan) and nuclei (magenta) (*scale bar*, 100 μm).

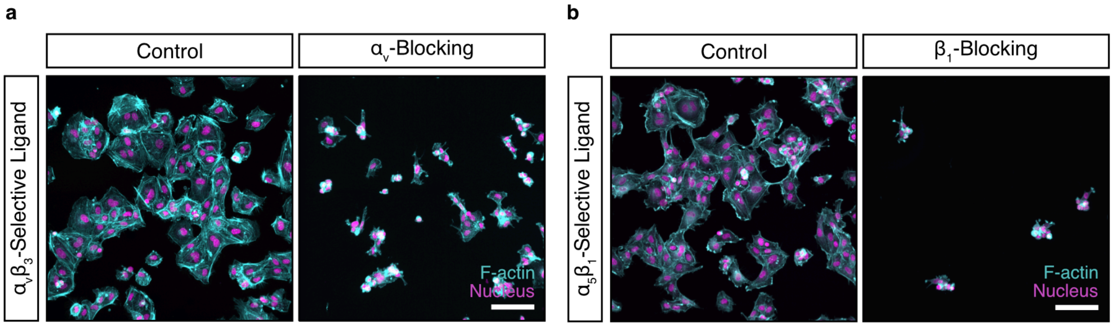

Supplementary Figure 9. Specificity of integrin $\alpha_5\beta_1$ and $\alpha_v\beta_3$-selective ligands. HUVECs were seeded on methacrylated dextran (DexMA) hydrogels functionalized with 1.5 mM $\alpha_v\beta_3$-selective ligand and 4.5 mM non-adhesive CGRGES peptide (**a**) or 1.5 mM $\alpha_5\beta_1$-selective ligand and 4.5 mM non-adhesive CGRGES peptide (**b**) and cultured in the presence of 20 µg/mL integrin $\alpha_v\beta_3$ blocking antibody (**a**) or 20 µg/mL integrin β_1 blocking antibody (**b**), compared to untreated controls. Composite fluorescence images of 3D projections showing F-actin (cyan) and nuclei (magenta) (*scale bar*, 100 µm).

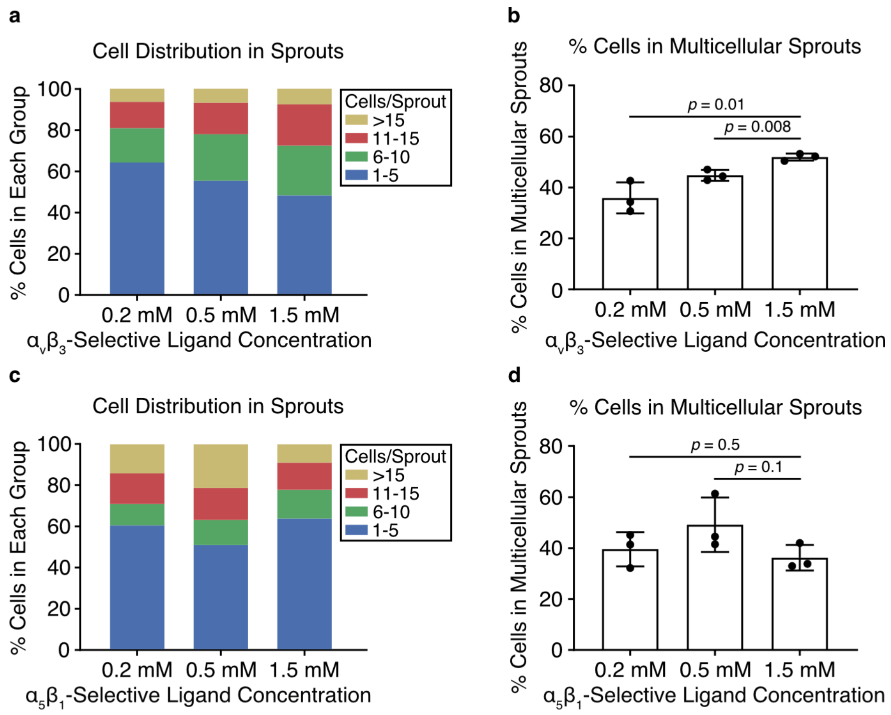

Supplementary Figure 10. Integrin $\alpha_v\beta_3$-selective ligand regulates angiogenic sprout multicellularity. Quantification of cell distribution across sprouts as a function of immobilized integrin $\alpha_v\beta_3$-selective ligand concentration (**a**) or integrin $\alpha_5\beta_1$-selective ligand concentration (**c**). Sprouts were divided into 4 groups according to cell number per sprout. **b, d** Quantification of sprout multicellularity (% cells in multicellular sprouts possessing 6 or more nuclei, relative to the total number of cells) (n = 3 independent experiments). All data presented as mean ± s.d., p < 0.05 is considered to be statistically significant (two-tailed unpaired Student's *t*-test). Source data are provided as a source data file.

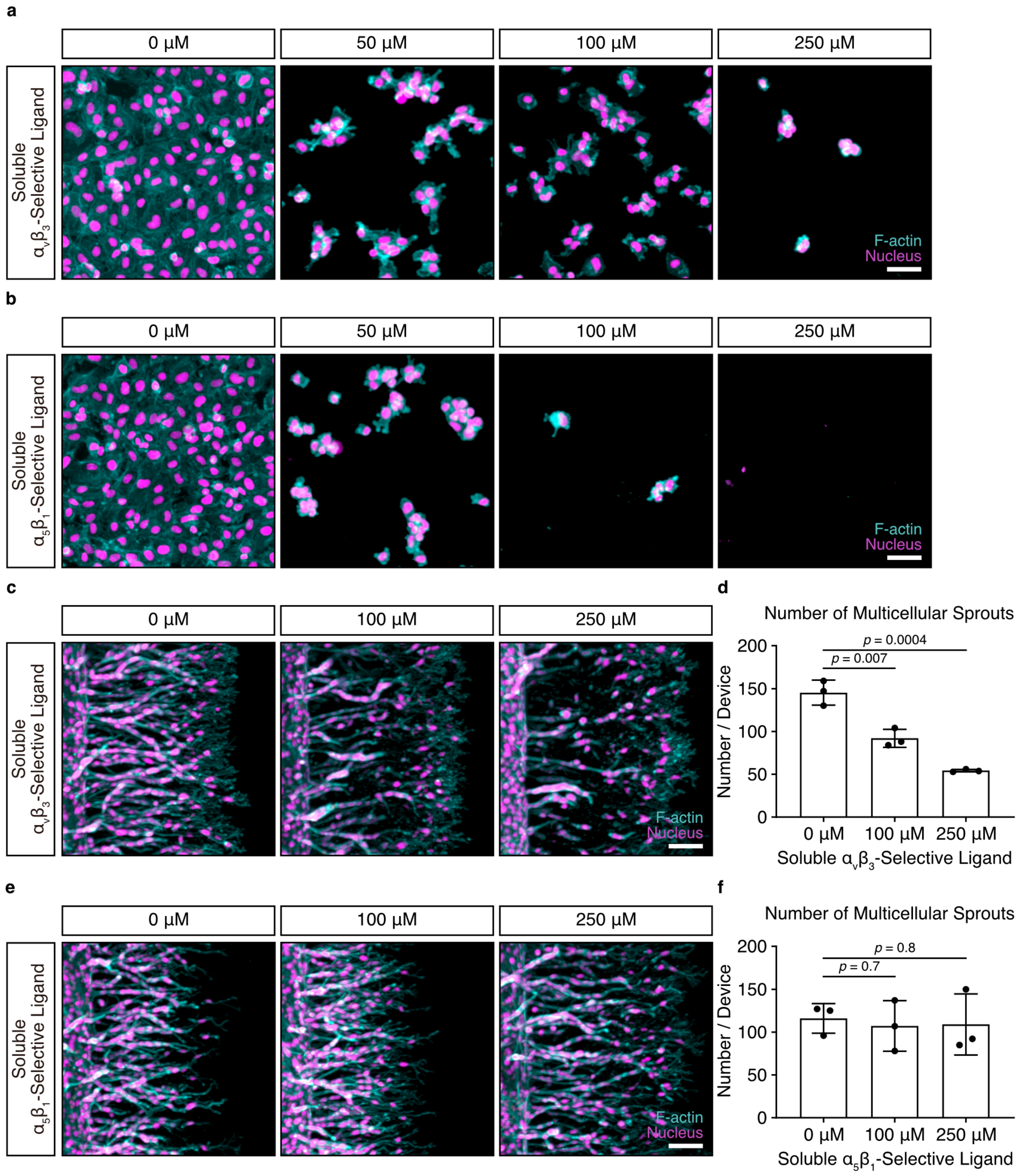

Supplementary Figure 11. Collective cell migration is inhibited by soluble integrin $\alpha_v\beta_3$-selective ligand. a, b HUVECs were seeded on methacrylated dextran (DexMA) hydrogels functionalized with 1.5 mM $\alpha_v\beta_3$-selective ligand and 4.5 mM non-adhesive CGRGES peptide (**a**) or 1.5 mM $\alpha_5\beta_1$-selective ligand and 4.5 mM non-adhesive CGRGES peptide (**b**) and cultured in the presence of soluble integrin $\alpha_v\beta_3$-selective ligand (**a**) or soluble integrin $\alpha_5\beta_1$-selective ligand (**b**). Composite fluorescence images of 3D projections showing F-actin (cyan) and nuclei (magenta)

(*scale bar*, 100 μm). **c, e** HUVECs invading into DexMA hydrogels functionalized with 6 mM immobilized CGRGDS, in the presence of varying concentrations of souble integrin $\alpha_v\beta_3$-selective ligand (**c**) or soluble integrin $\alpha_5\beta_1$-selective ligand (**e**) serving as integrin inhibitor. **d, f** Quantification of number of multicellular sprouts (sprouts per device possessing 6 or more nuclei) (n = 3 independent experiments). Composite fluorescence images of 3D projections showing F-actin (cyan) and nuclei (magenta) (*scale bar*, 100 μm). All data presented as mean ± s.d., $p < 0.05$ is considered to be statistically significant (two-tailed unpaired Student's *t*-test). Source data are provided as a source data file.

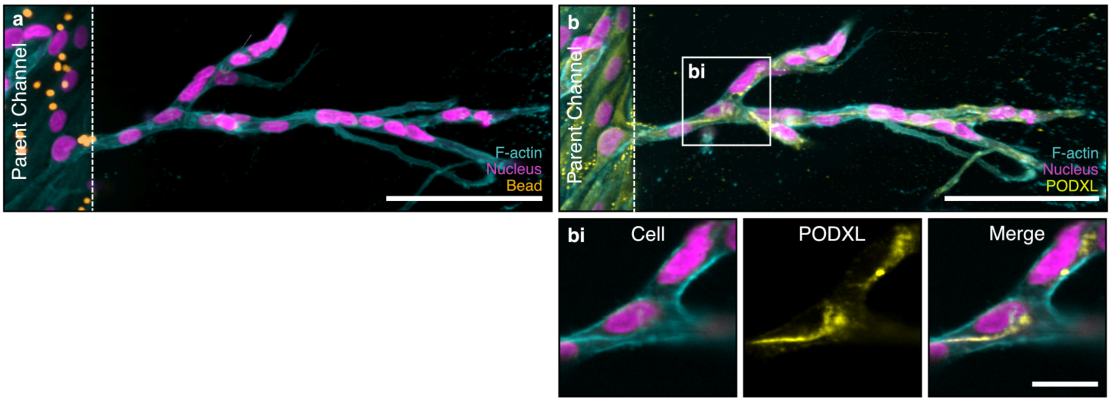

Supplementary Figure 12. Lumen formation is not yet initiated during collective migration of endothelial cells. HUVECs invading into a dextran vinyl sulfone (DexVS) hydrogel functionalized with 12 mM CGRGDS and crosslinked with 25.2 mM peptide of native collagen-derived degradability (NCD) for 4 days. At early timepoints, cells migrate collectively, whereas lumens have not formed yet. **a** 4 μm diameter fluorescent beads (yellow) added from the parent channel cannot enter the sprouts (*scale bar*, 100 μm). **b** Apical-basal polarity is being established in early sprouts, as visualized by the discontinuous localization of the luminal marker podocalyxin (PODXL, yellow) (*scale bar*, 100 μm). **bi** Horizontal section of the sprout to show the localization of podocalyxin (*scale bar*, 20 μm).

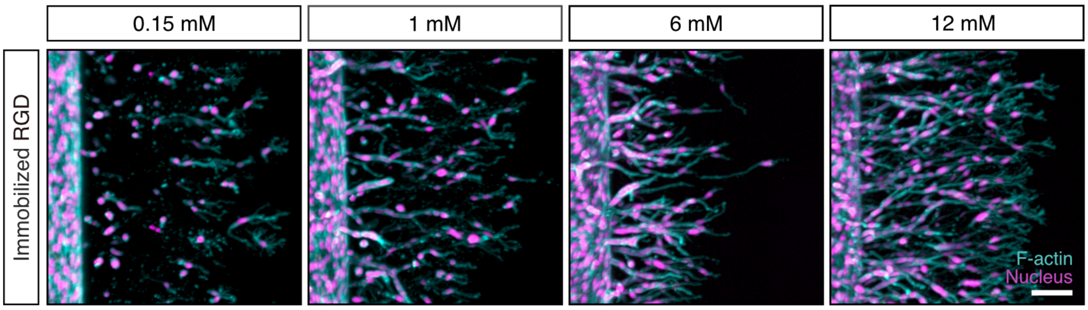

Supplementary Figure 13. Matrix adhesiveness regulates multicellularity of angiogenic sprouting in dextran vinyl sulfone (DexVS) hydrogels. HUVECs invading DexVS hydrogels functionalized with varying concentrations of the adhesive peptide CGRGDS for 4 days. Coupled ligand concentration was kept constant at 12 mM by adjusting with non-adhesive ligand CGRGES. Concentration of native collagen-derived degradability (NCD) crosslinker was kept constant at 25.2 mM to ensure comparable stiffness. Composite fluorescence images of 3D projections showing F-actin (cyan) and nuclei (magenta) (*scale bar*, 100 μm).

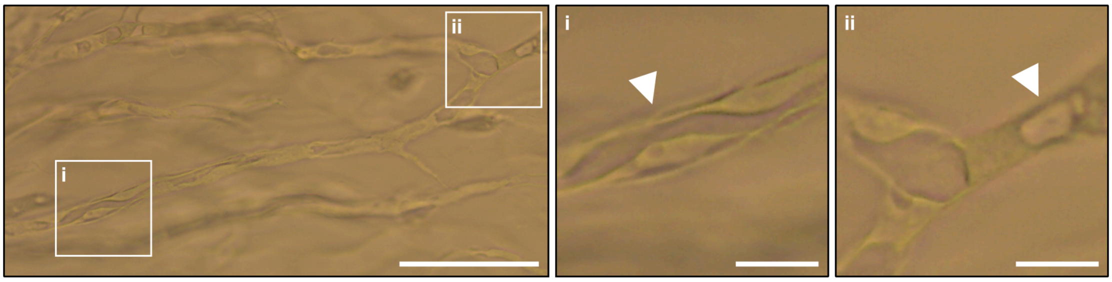

Supplementary Figure 14. Visualization of extracellular lumens and intracellular vacuoles by brightfield microscopy. HUVECs invading into a dextran vinyl sulfone (DexVS) hydrogel functionalized with 12 mM CGRGDS and crosslinked with 25.2 mM peptide of native collagen-derived degradability (NCD) for 14 days (*scale bar*, 100 µm, insets i and ii 20 µm).

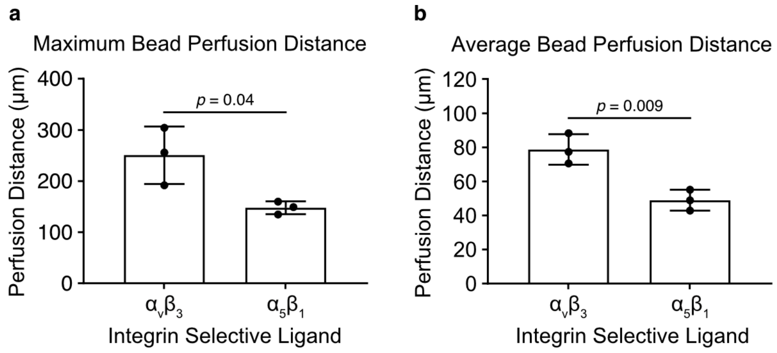

Supplementary Figure 15. Characterization of lumen formation in hydrogels functionalized with integrin $\alpha_v\beta_3$- and integrin $\alpha_5\beta_1$-selective ligands. Quantification of maximum bead perfusion distance (**a**) and average bead perfusion distance (**b**) in hydrogels functionalized with 1.5 mM integrin $\alpha_v\beta_3$-selective ligand or 1.5 mM integrin $\alpha_5\beta_1$-selective ligand, relative to lumen opening position at parent channel (n = 3 independent experiments). All data presented as mean ± s.d., $p < 0.05$ is considered to be statistically significant (two-tailed unpaired Student's *t*-test). Source data are provided as a source data file.

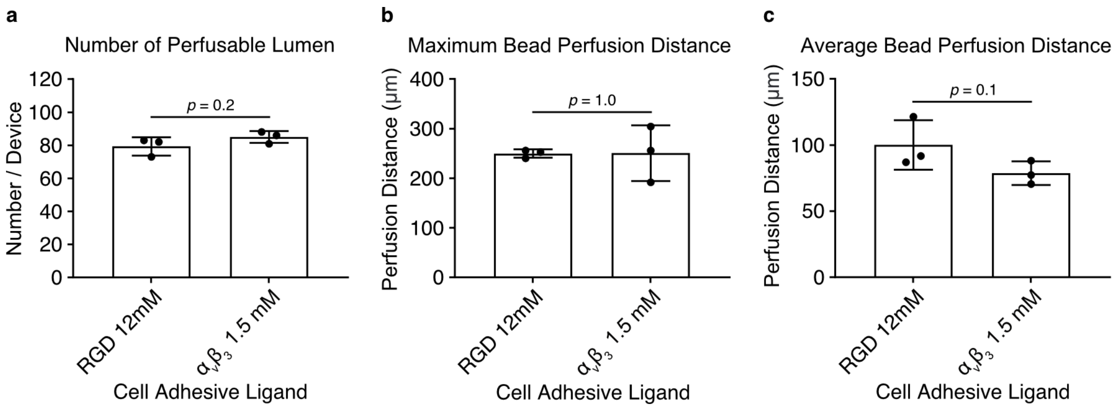

Supplementary Figure 16. Comparison of lumen formation in hydrogels functionalized with CGRGDS or integrin $\alpha_v\beta_3$-selective ligand. Quantification of number of perfusable lumens (**a**), maximum bead perfusion distance (**b**) and average bead perfusion distance (**c**) in hydrogels functionalized with 12 mM RGD or 1.5 mM integrin $\alpha_v\beta_3$-selective ligand (n = 3 independent experiments). All data presented as mean ± s.d., $p < 0.05$ is considered to be statistically significant (two-tailed unpaired Student's *t*-test). Source data are provided as a source data file.

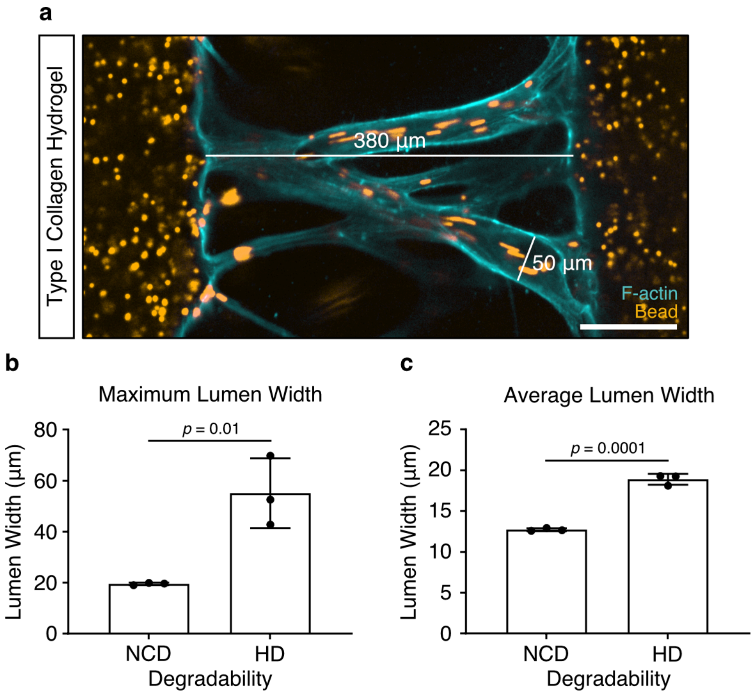

Supplementary Figure 17. Comparison of lumen formation in hydrogel of natural and synthetic origin. a HUVECs were induced to sprout into a 2.5 mg/mL type I collagen hydrogel for 9 days. Composite fluorescence images of 3D projections showing F-actin (cyan) and fluorescent beads (yellow) (*scale bar*, 100 µm). Quantification of maximum (**b**) and average (**c**) lumen width in hydrogels crosslinked with 25.2 mM native collagen-derived degradability (NCD) or high degradability (HD) peptides (n = 3 independent experiments). All data presented as mean ± s.d., $p < 0.05$ is considered to be statistically significant (two-tailed unpaired Student's *t*-test). Source data are provided as a source data file.

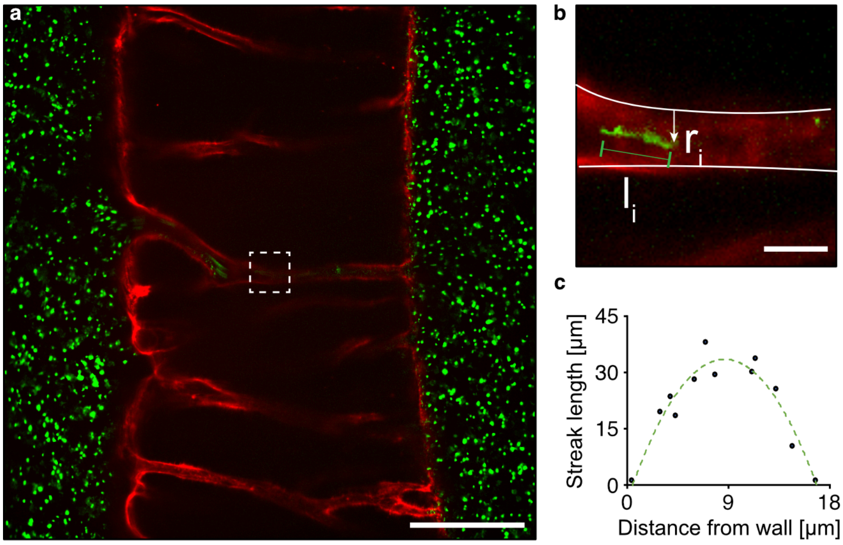

Supplementary Figure 18. Measurement of flow characteristics within neovessels. a 1 µm diameter fluorescent beads were introduced to characterize fluid velocities under defined fluid pressures (green – fluorescent beads, red – ECs, scale bar 150 µm). **b** Streaks of flowing fluorescent beads from an ROI (indicated by dashed line) were captured by opening the camera shutter on a spinning disk confocal for defined times. The length of each streak (**l_i**) and the distance of the origin of the streak from the vessel wall (**r_i**) was measured from resulting images (*scale bar*, 20 µm). **c** Plotting streak length as a function of distance from the wall shows the characteristic parabolic flow profile of Poiseuille flow.

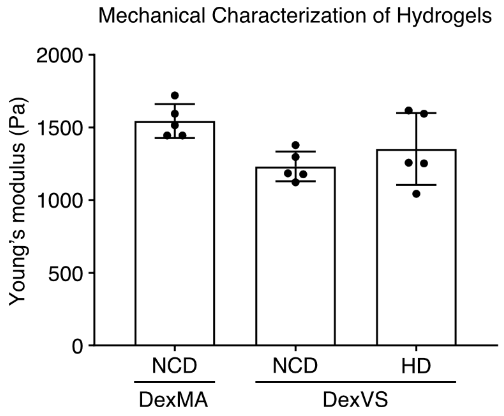

Supplementary Figure 19. Mechanical characterization of hydrogels. Young's modulus of methacrylated dextran (DexMA) hydrogels crosslinked with 30.5 mM native collagen-derived degradability (NCD) peptide and dextran vinyl sulfone (DexVS) hydrogels crosslinked with 25.2 mM NCD or high degradability (HD) peptide. All data presented as mean ± s.d.. Source data are provided as a source data file.
