# Supplementary material for: Synthetic extracellular matrices with tailored adhesiveness and degradability support lumen formation during angiogenic sprouting
Source: Nat Commun. 2021 Jun 7;12:3402. doi: 10.1038/s41467-021-23644-5 (PMC8184799; doi:10.1038/s41467-021-23644-5)
Supplement: Supplementary file 3 — Description of Additional Supplementary Files [file 41467_2021_23644_MOESM3_ESM.docx]

Description of Additional Supplementary Files

Title: Supplementary Movie 1.

Description: Neovessels connecting parent and growth factor source channels are perfusable. HUVECs were induced to sprout into a dextran vinyl sulfone (DexVS) hydrogel functionalized with 12 mM CGRGDS and crosslinked with 25.2 mM peptide of high degradability (HD) for 21 days. Perfusion was visualized with 1 μm diameter fluorescent beads (yellow) added from the growth factor source channel. Composite fluorescence images of 3D projections showing F-actin (cyan) (scale bar, 100 μm).
