# Supplementary material for: Synthetic extracellular matrices with tailored adhesiveness and degradability support lumen formation during angiogenic sprouting
Source: Nat Commun. 2021 Jun 7;12:3402. doi: 10.1038/s41467-021-23644-5 (PMC8184799; doi:10.1038/s41467-021-23644-5)
Supplement: Supplementary file 5 — Reporting Summary [file 41467_2021_23644_MOESM5_ESM.pdf]

nature research

Corresponding author(s): Britta Trappmann

Last updated by author(s): 22.04.2021

Reporting Summary

Nature Research wishes to improve the reproducibility of the work that we publish. This form provides structure for consistency and transparency in reporting. For further information on Nature Research policies, see our Editorial Policies and the Editorial Policy Checklist.

Statistics

For all statistical analyses, confirm that the following items are present in the figure legend, table legend, main text, or Methods section.

n/a	Confirmed	
☐	☒	The exact sample size (n) for each experimental group/condition, given as a discrete number and unit of measurement
☐	☒	A statement on whether measurements were taken from distinct samples or whether the same sample was measured repeatedly
☐	☒	The statistical test(s) used AND whether they are one- or two-sided<br>*Only common tests should be described solely by name; describe more complex techniques in the Methods section.*
☒	☐	A description of all covariates tested
☒	☐	A description of any assumptions or corrections, such as tests of normality and adjustment for multiple comparisons
☐	☒	A full description of the statistical parameters including central tendency (e.g. means) or other basic estimates (e.g. regression coefficient) AND variation (e.g. standard deviation) or associated estimates of uncertainty (e.g. confidence intervals)
☐	☒	For null hypothesis testing, the test statistic (e.g. F, t, r) with confidence intervals, effect sizes, degrees of freedom and P value noted<br>*Give P values as exact values whenever suitable.*
☒	☐	For Bayesian analysis, information on the choice of priors and Markov chain Monte Carlo settings
☒	☐	For hierarchical and complex designs, identification of the appropriate level for tests and full reporting of outcomes
☒	☐	Estimates of effect sizes (e.g. Cohen's d, Pearson's r), indicating how they were calculated

Our web collection on statistics for biologists contains articles on many of the points above.

Software and code

Policy information about availability of computer code

Data collection	Confocal imaging: Fusion, version 2.0.0.13; brightfield imaging: Leica application suite, version 3.4.0
Data analysis	Data were analyzed using Sigmaplot for Windows, version 14.0; GraphPad Prism, Version 9.0.0; ImageJ, version 2.0.0-rc-68/1.52e; Microsoft Excel for Mac, version 16.30 (19101301); IMARIS, version x64, 9.5.1.

For manuscripts utilizing custom algorithms or software that are central to the research but not yet described in published literature, software must be made available to editors and reviewers. We strongly encourage code deposition in a community repository (e.g. GitHub). See the Nature Research guidelines for submitting code & software for further information.

Data

Policy information about availability of data

All manuscripts must include a data availability statement. This statement should provide the following information, where applicable:

- Accession codes, unique identifiers, or web links for publicly available datasets
- A list of figures that have associated raw data
- A description of any restrictions on data availability

The data sets generated and analyzed during this study are available from the corresponding author upon reasonable request. A reporting summary for this article is available as a Supplementary Information file. Source data are provided with this paper.

Field-specific reporting

Please select the one below that is the best fit for your research. If you are not sure, read the appropriate sections before making your selection.

[x] Life sciences [ ] Behavioural & social sciences [ ] Ecological, evolutionary & environmental sciences

For a reference copy of the document with all sections, see nature.com/documents/nr-reporting-summary-flat.pdf

Life sciences study design

All studies must disclose on these points even when the disclosure is negative.

Sample size	Sample size calculation was not performed prior to the experiments. A sample size of three biologically independent replicates was chosen to validate reproducibility of the results, based on standards established in the field, including a previous publication from our group (Trappmann et al., Nature Communications 2017).
Data exclusions	No data were excluded from analysis.
Replication	All experiments were repeated three independent times. Most importantly, the experimental outcomes between independent experiments were in all cases comparable.
Randomization	Randomization was not required for this study. Microfluidic devices were assembled and randomly assigned to be filled with hydrogels of different compositions. Endothelial cell seeded device were randomly assigned to the different control/inhibitor treatment groups.
Blinding	Blinding was not applicable to this study, since for all statistical analyses, cells and sprouts in entire samples were counted (no picking of individual cells or sprouts).

Reporting for specific materials, systems and methods

We require information from authors about some types of materials, experimental systems and methods used in many studies. Here, indicate whether each material, system or method listed is relevant to your study. If you are not sure if a list item applies to your research, read the appropriate section before selecting a response.

Materials & experimental systems

n/a	Involved in the study	
[ ]	[x]	Antibodies
[ ]	[x]	Eukaryotic cell lines
[x]	[ ]	Palaeontology and archaeology
[ ]	[x]	Animals and other organisms
[x]	[ ]	Human research participants
[x]	[ ]	Clinical data
[x]	[ ]	Dual use research of concern

Methods

n/a	Involved in the study	
[x]	[ ]	ChIP-seq
[x]	[ ]	Flow cytometry
[x]	[ ]	MRI-based neuroimaging

Antibodies

Antibodies used	Primary antibodies:<br>Mouse anti-human podocalyxin: R&D Systems, MAB1658, lot number JKW0219011<br>Rabbit anti-human collagen IV: Abcam, ab6586, lot number GR3317997<br>Mouse anti-integrin αvβ3 antibody, clone LM609, Merck Chemicals, MAB1976Z, lot number: 2814858<br>Rabbit anti-laminin, rabbit anti-laminin 411, rabbit anti-laminin 511 and mouse anti-integrin β1, clone P5D2, were prepared by the laboratory of Lydia Sorokin, University of Muenster, Germany.<br><br>Secondary antibodies:<br>Alexa Fluor 555 donkey anti-mouse IgG, Thermo Fisher, A-31570, lot number 1774719<br>Alexa Fluor 647 goat anti-rabbit IgG, Thermo Fisher, A-21244, lot number 2134003
Validation	Rabbit anti-laminin 411, rabbit anti-laminin 511 and rabbit anti-laminin antibodies: Sixt, M. et al., J Cell Biol 153, 933-945 (2001).<br>Mouse anti-integrin β1 antibody: Seltzer et al., Exp Cell Res 213, 365-374 (1994) and Wayner and Carter, J Cell Biol 105, 1873-1884 (1987).<br>Mouse anti-integrin αvβ3 antibody: validated by Merck Chemicals GmbH (https://www.sigmaaldrich.com/catalog/product/mm/mab1976z?lang=de&region=DE)<br>Rabbit anti-human collagen IV antibody: validated by Abcam (https://www.abcam.com/collagen-iv-antibody-ab6586.html)<br>Mouse anti-human podocalyxin antibody: validated by R&D Systems (https://www.rndsystems.com/cn/products/human-podocalyxin-antibody-222328_mab1658#product-citations)

All commercial antibodies have been used in numerous literature reports.

Eukaryotic cell lines

Policy information about cell lines

Cell line source(s)	Human umbilical cord vein endothelial cells (HUVECs) and human lung microvascular endothelial cells (HMVECs-L) were purchased from Lonza.
Authentication	Cells were used as received, certificate of analysis provided by Lonza. Both HUVECs and HMVECs were characterized to be CD31 positive by flow cytometry.
Mycoplasma contamination	All cells tested negative for mycoplasma contamination.
Commonly misidentified lines (See ICLAC register)	In this study, no commonly misidentified cell lines were used.

Animals and other organisms

Policy information about studies involving animals; ARRIVE guidelines recommended for reporting animal research

Laboratory animals	10 week-old C57/BL6 wild-type (Charles River Laboratories), female
Wild animals	No wild animals were used in this study.
Field-collected samples	No field-collected samples were used in this study.
Ethics oversight	Animal experiments and husbandry were performed according to the German Animal Welfare guidelines and approved by the Landesamt für Natur, Umwelt und Verbraucherschutz Nordrhein-Westfalen (State Agency for Nature, Environment and Consumer Protection of North Rhine-Westphalia).

Note that full information on the approval of the study protocol must also be provided in the manuscript.
